# Adaptation and validation of the Multidimensional Measure of Parasocial Relationships (MMPR) in Poland

**DOI:** 10.1038/s41598-025-11666-8

**Published:** 2025-07-17

**Authors:** Aleksandra Witkowska, Dorota Mącik, Danilo Garcia

**Affiliations:** 1 Department of Clinical Psychology, John Paul II Catholic University of Lublin, Lublin, Poland; 2 Department of Social Studies, University of Stavanger, Stavanger, Norway; 3 Department of Behavioral Sciences and Learning, Linköping University, Linköping, Sweden; 4 Department of Social Studies, Promotion of Health and Innovation for Well-Being (PHI-WELL), University of Stavanger, Stavanger, Norway; 5 International Network for Well-Being, Lab for Biopsychosocial Personality Research (BPS-PR), Stavanger, Norway; 6 International Network for Well-Being, Promotion of Health and Innovation (PHI) Lab, Linköping, Sweden; 7 Centre for Ethics, Law and Mental Health (CELAM), University of Gothenburg, Gothenburg, Sweden; 8 Department of Psychology, University of Gothenburg, Gothenburg, Sweden

**Keywords:** Parasocial relationships, Social media, Psychometric validation, Cross-cultural adaptation, Poland, Psychology, Human behaviour

## Abstract

Parasocial relationships, one-sided bonds with media figures, have grown with Internet/social media use and are linked to various well-being outcomes. For example, parasocial relationships on social media may foster connection and healthy behaviors while also prompting negative self-comparisons. The Multidimensional Measure of Parasocial Relationships (MMPR), developed by Garcia and colleagues (2022), assess parasocial engagement across affective, behavioral, cognitive, and decisional dimensions. While the MMPR has demonstrated robust psychometric properties in its original Swedish version, its cross-cultural applicability remains unexplored. To addresses this gap, we adapted and validated the MMPR in a Polish sample. A total of 371 Polish young adults (255 women, 116 men; age range 18–48 years) completed the survey. The adaptation process involved translation, back-translation, and review by expert judges. Confirmatory factor analysis (CFA) tested the four-factor model. We also calculated Internal consistency (Cronbach’s alpha and McDonald’s omega), four-week test–retest reliability, and examined convergent validity via correlations with theoretically related measures (i.e., early maladaptive schemas and emotional well-being). CFA supported the four-dimensional structure: χ^2^/df = 2.74; RMSEA = .069 (90% CI = .060–.077); SRMR = .069; CFI = .855. Cronbach’s α ranged between .59 (Behavioral dimension) and .75 (Decisional dimension) (.83 for the whole scale). Test–retest correlations were moderate to strong (*r* = .47-.81). Convergent validity revealed expected, but weak, associations (e.g., higher parasocial engagement was linked to lower abandonment schemas and lower negative emotions). Our findings support the MMPR as a psychometrically sound instrument for assessing parasocial relationships in a digital Polish context. Despite limitations such as the sample’s demographic composition and modest internal consistency for the Behavioral subscale, the study contributes meaningful evidence for the scale’s applicability beyond its original cultural setting. The results underscore the importance of culturally adapted tools to capture the complex interplay between media engagement and psychosocial functioning, particularly in an era of increasing online interaction. Future research should further refine the measure and explore its use across diverse populations and platforms.

## Introduction

In the twenty-first century, social media platforms have amplified the influence of public figures on individuals’ thoughts, emotions, and decision-making to unprecedented levels. One outcome of this media-saturated environment is the rise of parasocial relationships, one-sided, non-reciprocal bonds people form with celebrities, influencers, or other media figures they follow online. These relationships are characterized by perceived intimacy and constant access to the public figure’s life through platforms such as Instagram, TikTok, or YouTube. Fans may feel a deep personal connection to a favorite influencer, even though the celebrity is typically unaware of their existence. Social media blurs the line between reality and illusion by enabling real-time interactions that create a impression of mutual engagement despite the fundamentally unidirectional nature of the exchange. It is worth noting the distinction between parasocial interactions and parasocial relationships, where the former refers to brief one-off interactions (e.g., commenting on a life stream) that not need to develop into a lasting sense of relationships, while the latter refers to relationships that can persist in the absence of direct interaction^1–3^.

Horton and Wohl^1^, who first conceptualized parasocial relationships, identified three core features that define these relationships: *friendship*, *understanding*, and *identification*. *Friendship* in this context refers to the audience’s perceived mutual connection with the media figure, a feeling of intimacy, affection, and even reciprocal appreciation, despite the relationship being entirely one-sided. This sense of friendship is cultivated through repeated exposure; over time, fans come to believe they share a close bond with the celebrity as they learn about the figure’s life and personality. *Understanding* denotes the fan’s belief they truly know the media figure on a personal level. Followers accumulate knowledge about the celebrity’s values, habits, and motivations, reinforcing an illusion of intimate familiarity. *Identification* involves internalizing the celebrity’s attitudes or behaviors and seeing the media figure as aspirational versions of oneself. Through identification, fans develop a psychological attachment and loyalty to the figure, adopting similar viewpoints or lifestyle choices in the process^4^. Together, these three features help explain how viewers can feel genuinely connected to someone who does not reciprocate that relationship.

Parasocial relationships are further reinforced by social-cognitive processes. Audiences actively attribute traits, motives, and emotions to the media personalities based on the content those figures share. For instance, a television presenter who consistently smiles and exudes positivity might be perceived as friendly and optimistic by viewers. In the digital era, influencers enhance this effect by sharing personal details in daily updates, behind-the-scenes moments, or engaging directly with followers in comments or live sessions, making themselves appear authentic and relatable. These perceptions build a sense of familiarity and trust. Individuals are more inclined to form and engage parasocial bonds with media figures whom they consider similar to themselves in values, beliefs, or experiences. When a public figure is seen as genuine and realistic in their self-presentation, it strengthens the audience’s feeling of closeness and emotional investment^5^. On the behavioral side, fans often express their parasocial engagement through action such as “liking”, sharing content, or commenting. Interactions that give a momentary illusion of mutual connection with the media figure, even though they address a mass audience^6^. Media figures encourage these behaviors by posting content that feels personal (e.g. behind-the-scenes updates, candid stories), which invites followers to respond and thus deepens the follower’s sense of involvement.

In this context, the formation and maintenance of parasocial bonds have been examined through multiple theoretical lenses. For instance, Cole and Leets^7^ explored the development of parasocial relationships through three key relational theories. The uncertainty reduction theory (I) posits that parasocial relationships evolve as uncertainty about the other person decreases, thus fostering trust and predictability. As viewers gain a better understanding of a celebrity’s behavior, their liking and attachment often increase. The personal construct theory (II) suggests that individuals use cognitive schemas and past interpersonal patterns to interpret and evaluate figures, creating a subjective sense of familiarity and knowledge. Additionally, social exchange theory (III) highlights the cost–benefit analysis in parasocial relationships, where users perceive high emotional rewards and low relational costs when engaging with media personalities. These theories collectively provide a framework for understanding the multidimensional formation and maintenance of parasocial relationships, particularly within online communities^8^.

Hence, understanding the multifaceted nature of parasocial relationships and their impact on individuals’ emotions, behaviors, and decisions requires robust measurement tools. The Multidimensional Measure of Parasocial Relationships (MMPR), developed by Garcia, Björk, and Kazemitabar^6^ (see Table 1), was specifically designed to address this need. Grounded in the multidimensional model of attitudes, the MMPR provides a comprehensive framework to assess the Affective, Behavioral, Cognitive, and Decisional dimensions of parasocial relationships. These dimensions reflect not only how individuals feel, think, and interact with media figures but also the extent to which media figures influence their daily decisions. The development of the MMPR involved rigorous psychometric evaluations, including exploratory factor analysis (EFA), confirmatory factor analysis (CFA), intern consistency testing, correlation analysis, and structural equation modeling (SEM). EFA supported a four-factor structure, and CFA identified the bifactor model with correlated factors as the best fit for the data, although adjustments to specific items, such as item 16 (”I happily follow different tips and advice that the social media figure shares because I feel I can trust his/her knowledge about these things.”) within the Decisional dimension, could further refine model fit. The MMPR demonstrated strong internal consistency, with Cronbach’s alpha values ranging from 0.66 for the Behavioral dimension to 0.75 for the Decisional dimension, and an overall alpha of 0.85. The more precise ordinal alpha values further underscored its reliability: 0.90 for Affective, 0.95 for Behavioral, 0.90 for Cognitive, and 0.88 for Decisional dimensions, with a robust 0.93 for the total score. Research utilizing the MMPR has shown that higher engagement in parasocial relationships is associated with lower self-esteem. This relationship is mediated by a positive relationship with social comparison, emphasizing the impact of these relationships on self-perception. These findings align with previous research^9–12^, thereby supporting further the MMPR’s validity and the multidimensionality of parasocial relationships. However, the MMPR has only been validated in the Swedish cultural context.

**Table 1:** The Multidimensional Measure of Parasocial Relationships (MMPR)^1^ in English and Polish.

	**English**	**Polish**	
**Instructions**	This questionnaire contains statements about your attitudes, that is, your thoughts, feelings, and behavior regarding a specific social media figure. Before giving your answers, please think of a specific social media figure, preferably the one that you follow the most. It can be an influencer, a youtuber, tiktoker, or a social media figure within, for example, lifestyle, exercise, sports, gaming, nutrition, or fashion. The most important thing is that you don’t have a relation with this person in real life Please answer to which degree you agree or disagree to the following statements:	Kwestionariusz ten zawiera stwierdzenia na temat Twoich postaw, czyli myśli, uczuć i zachowań w odniesieniu do konkretnej postaci w mediach społecznościowych. Zanim udzielisz odpowiedzi, pomyśl o konkretnej osobie z mediów społecznościowych, najlepiej tej, którą najczęściej obserwujesz. Może to być influencer, youtuber, tiktoker lub postać z mediów społecznościowych, na przykład zajmująca się stylem życia, ćwiczeniami, sportem, grami, odżywianiem lub modą. Najważniejsze jest to, że nie masz z tą osobą relacji w prawdziwym życiu Odpowiedz, w jakim stopniu zgadzasz się lub nie zgadzasz z poniższymi stwierdzeniami	**Instrukcje**
**Affective dimension**	I experience a feeling of connectedness with the media figure through his/her posts on social media	*Czuję więź z osobą medialną poprzez jego/jej posty w mediach społecznościowych*	**Wymiar afektywny**
	I experience emotional engagement when the social media figure shares more private information about himself/herself (e.g., bigger life events)	Doświadczam, że angażuję się emocjonalnie, gdy osoba medialna dzieli się bardziej prywatnymi informacjami na swój temat	
	I don’t feel like I can personally relate to the content in the social media figure´s posts	Uważam, że osobiście nie utożsamiam się z treścią postów osoby medialnej	
	I often feel that I get inspired by the social media figure´s posts	Często czuję się zainspirowany postami publikowanymi przez osobę medialną	
**Cognitive dimension**	I think that the social media figure represents values that are important to me	Zawsze “lajkuję” posty osoby medialnej w mediach społecznościowych	**Wymiar poznawczy**
	I don’t think that the media figure portrays himself/herself in an authentic way on social media	Często komentuję posty osoby medialnej w polu komentarzy	
	I see most of what the media figure shares on social media in a positive way	Często przesyłam posty osoby medialnej moim znajomym lub udostępniam je na własnych kanałach internetowych	
	The social media figure seems to be a genuine person that I would get along with in real life	Przeważnie sprawdzam tylko, co nowego opublikowała osoba medialna i nie jestem aż tak aktywny, jeżeli chodzi o klikanie “lubię to”, udostępnianie czy komentowanie	
**Behavior dimension**	I always “like” the media figure’s posts on social media	Uważam, że osoba medialna reprezentuje wartości, które są dla mnie ważne	**Wymiar behawioralny**
	I often comment on the social media figure´s posts in the comment field	Nie sądzę, aby osoba medialna przedstawiała się w autentyczny sposób w mediach społecznościowych	
	I often forward the social media figure´s posts to my friends or share them on my own online feeds	Większość tego, co osoba medialna udostępnia w mediach społecznościowych, oceniam pozytywnie	
	I mostly just check the social media figure´s updates and am not that active with liking, sharing, or commenting	Osoba medialna wydaje się autentyczną osobą, z którą dogadałbym się w prawdziwym życiu	
**Decisional dimension**	I prefer things that the social media figure is marketing (e.g., products, nutrition advice, training advice, etc.) over similar things that are marketed in other places	Wolę rzeczy, które osoba medialna promuje (np. produkty, porady żywieniowe, porady dotyczące szkoleń itp.), niż podobne rzeczy reklamowane w innych miejscach	**Wymiar decyzyjny**
	The social media figure´s posts often inspire me to make changes in my own life	Posty osoby medialnej często inspirują mnie do zmian we własnym życiu	
	I never buy products that the media figure is marketing or giving advice about on social media	Nigdy nie kupuję produktów, które osoba medialna reklamuje lub poleca w mediach społecznościowych	
	I happily follow different tips and advice that the social media figure shares because I feel I can trust his/her knowledge about these things	Chętnie stosuję się do różnych wskazówek i rad, którymi dzieli się osoba medialna, ponieważ czuję, że mogę zaufać jego wiedzy na ten temat	
	It often happens that, in conversations with other people in my everyday life, I point out things that the media figure has mentioned in his/her posts on social media	Często zdarza się, że w rozmowach z innymi osobami na co dzień zwracam uwagę na rzeczy, o których osoba medialna wspomniała w swoich postach w mediach społecznościowych	
	There are times when the social media figure´s posts contribute to me changing my lifestyle in some way (e.g., clothing, diet, training routine, looks, and etc.)	Zdarza się, że posty osoby medialnej przyczyniają się do tego, że w jakiś sposób zmieniam swoje przyzwyczajenia (np. ubiór, dietę, treningi, wygląd itp.)	

Note: Originally published in Björk, E. (2021). Parasociala relationer som attityd till mediaprofiler i sociala Medier – Komponenter i attitydbildning och dess samband med social jämförelse och självkänsla [Bachelor Thesis, Linköping University]. See: Garcia, D., Björk, E., & Kazemitabar, M. (2022). The A(ffect) B(ehavior) C(ognition) D(ecision) of Parasocial Relationships: A pilot study on the psychometric properties of the multidimensional measure of parasocial relationships (MMPR). Heliyon, 8:e10779. https://doi.org/10.1016/j.heliyon.2022.e10779. Translation to polish by Dorota Mącik, Aleksandra Witkowska, and Aleksandra Wójtowicz (2024). For any use, research or commercial, please contact elina.bjork@icloud.com and danilo.garcia@icloud.com.

Parasocial relationships are multifaceted in their outcomes as well, capable of conferring benefits but also posing risks for individual well-being. On the positive side, parasocial bonds can provide a sense of social support and companionship, particularly for individuals who experience loneliness, social isolation, or a lack of close real-world relationships. Engaging with a favorite media figure can offer emotional comfort and opportunities for second-hand emotional expression, which may bolster psychological resilience and contribute to improved well-being. Moreover, media figures often serve as role models or sources of inspiration. By observing and admiring influencer’s attitudes and behaviors, fans might adopt healthier habits or more positive outlooks; for example influencers who speak openly about mental health or personal challenges can reduce stigma and encourage followers’ personal growth. Parasocial engagement can also foster a sense of community belonging. Fans who follow and interact around the same celebrity may feel part of an implicit community, and exposure to diverse media figures can even diminish prejudice, a phenomenon akin to an online parasocial contact hypothesis, wherein seeing the world through a beloved figure’s perspective increases empathy, inclusivity, and acceptance of others^13^.

On the other hand, excessive or maladaptive engagement in parasocial relationships may have adverse consequences. Some individuals withdraw from real-life social interactions in favor of online engagement, immersing themselves in parasocial relationships or virtual communities to the extent that interferes with their offline life. This preference for online socialization over face-to-face interaction can be a contributing factor to problematic Internet use^14^. For example, a meta-analysis showed that excessive Internet use has a significant negative impact on overall well-being, encompassing lower life satisfaction, compromised psychological well-being, and reduced self-esteem^15^. Similarly, recent research^16^ has found negative associations between problematic Internet use and various quality-of-life indicators (e.g., daily functioning, life satisfaction, and the psychological dimension of quality of life). Thus, an overreliance on one-sided online connections, including parasocial bonds, may coincide with declines in mental health and happiness. Furthermore, parasocial attachments can lead to neglect of real-world relationships. If someone comes to depend on social media and celebrity content as their primary source of emotional fulfillment, they might invest less effort in reciprocal, offline relationships. This displacement can erode social skills or deepen feelings of isolation in the long run. Another potential pitfall is the idealization of media figures. Celebrities often curate the best parts of their lives online, which can set unrealistically high expectations for what relationships or life in general should be like. Followers who compare their own lives to these idealized portrayals may experience dissatisfaction or feelings of inadequacy^17^. When the realities of ordinary relationships don’t measure up to the seemingly perfect connection a fan imagines with a celebrity, this disillusionment can increase social disconnection and unhappiness. In extreme cases, devotion to a parasocial bond can even become obsessive, crowding out other activities or relationships, a pattern noted in studies of celebrity worship and Internet addiction. Balancing these perspectives, it is also recognized that not all Internet use or parasocial engagement is detrimental. Moderate, intentional use of online platforms can have a practical and well-being benefits. For instance, using the Internet to access useful information, self-improvement resources, or to maintain long-distance friendships has been associated with greater efficiency in daily tasks, personal development opportunities, and improved quality of life and well-being^18^. In summary, parasocial relationships can yield both positive psychosocial benefits and negative consequences, depending on how individuals engage with them and in what context. This duality underscores the need to better understand who engages in parasocial bonds and how such engagement relates to well-being and other psychological factors.

One particularly salient aspect of parasocial relationships engagement is its emotional component. By their nature, parasocial relationships involve a great deal of emotional investment from the fan’s side. Researchers have noted that both positive and negative emotions serve distinct functions in strengthening these one-sided bonds. When media figures share positive emotions, such as joy, excitement, and enthusiasm, it tends to foster approachability and warm, making followers more drawn to them. Expressing positive emotions increases an influencer’s appeal and helps audiences feel a joyful connection, thereby facilitating the formation of parasocial bond. In contrast, when media figures open up about negative emotions or personal struggles (e.g., sadness, anger, frustration), it can deepen fan’s sense of trust and intimacy. The willingness of a public figure to display vulnerability signals authenticity and sincerity, which fans often respond to with increased loyalty and feeling of closeness. In traditional interpersonal relationships, sharing negative emotions is a hallmark of close friendship or intimacy, and similarly, in parasocial contexts it can strengthen the perceived bond by making the relationship feel more “real” and safe for the fan. However, this dynamic hinges on authenticity. Inauthentic or contrived emotional displays, for instance, a virtual influencer (i.e., a computer-generated persona) feigning sadness, may create a sense of dissonance and actually weaken the parasocial connection^19^. Audiences are adept at sensing when a media personality’s emotional expression is genuine, and violations of that trust can lead to cynicism or disengagement. These insights into emotional exchange highlight why an individual’s own emotional well-being might be intertwined with parasocial engagement. If parasocial interactions provide a outlet for feeling joy or a comfort for dealing with sadness, one might expect that a person’s levels of positive and negative affect are related to the intensity of their parasocial relationships. Accordingly, the present study included measures of emotional well-being (both positive and negative affective experiences) as part of the convergent validity assessment, to examine how parasocial relationship engagement correlates with individuals’ emotional states.

In additional to emotional factors, certain underlying cognitive–behavioral patterns may predispose individuals to seek out parasocial connections. In particular, the concept of early maladaptive schemas offer a theoretical framework for understanding why some people might favor the safer, controlled world of parasocial relationships over traditional relationships. Early maladaptive schemas are broad, enduring negative patterns in thinking and feeling about oneself and one’s relationships, typically formed in childhood or adolescence due to unmet emotional needs or adverse experiences^20^. These schemas shape how people interpret social situations and regulate their emotions^21,22^. For example, a person with a strong *abandonment* schema lives with an intense fear that loved ones will desert them; similarly, someone high on *defectiveness/shame* believes they are fundamentally unlovable or will be harshly judged if their true self is revealed. Such individuals often experience anxiety in interpersonal contexts, expecting rejection or humiliation. When an activating event occurs; say, being excluded by peers or experiencing a breakup, it triggers acute negative emotions, like loneliness, panic, or shame, that are difficult to cope with. To protect themselves, individuals with these schemas may try to avoid situations that could lead to rejection or emotional pain, which often means withdrawing from face-to-face social interactions^23^. Parasocial relationships can become an appealing alternative for these individuals. Engaging with a media figure provides a sense of connection and social safety without the fear of abandonment or ridicule, since the relationship does not require the person to risk vulnerability with a real partner. In essence, parasocial bonds offer control because the fan can engage or disengage on their own terms, and the media figure will never directly reject them. Consistent with this reasoning, problems in real-life interpersonal relationships have been empirically associated with higher endorsement of certain maladaptive schemas, specifically those related to disconnection and rejection.

Five early maladaptive schemas in particular, often categorized in the “disconnection/rejection” domain, stand out: emotional deprivation (the expectation that one’s need for affection will never be met), abandonment/instability (the expectation that relationships are fragile and people will leave), defectiveness/shame (the feeling of being flawed and unworthy of love), social isolation (feeling fundamentally alone or different from others), and mistrust/abuse (expectation that others will hurt or take advantage). Individuals who strongly harbor these schemas tend to struggle maintaining healthy relationships, often feeling insecure or anxious with others^24,25^. As a result, they may preferentially turn to alternative, non-reciprocal relationships that do not require direct interaction in the real world. Parasocial relationships and other online relationships fit this description, as they allow one-sided engagement where the person has greater control over the intensity and timing of interaction, and no risk of personal rejection. In line with previous research on interpersonal difficulties, we focused on these five schemas as potentially relevant to parasocial engagement, while excluding other schemas (such as *approval-seeking* or *unrelenting standards perfectionism*) that are less directly tied to attachment and were not found to be significantly related to interpersonal problems in prior studies^26,27^. By examining these cognitive schemas alongside parasocial relationship measures, the study can test whether those who form strong parasocial ties indeed exhibit the predicted underlying social-cognitive vulnerabilities. The inclusion of both emotional well-being indicators and maladaptive schema measures for convergent validity is intended to provide a richer understanding of how parasocial relationship tendencies connect with users’ psychological profiles. We anticipated, for instance, that higher parasocial engagement would correlate with higher levels of the five aforementioned schemas (i.e., indicating more interpersonal insecurities) and with particular patterns of emotional experience (e.g., possibly higher loneliness or negative affect, although parasocial comfort might also associate with slightly lower distress, as discussed earlier). Clarifying these links is not only theoretically interesting but also important for assessing the construct validity of how researchers measure parasocial relationships in different cultural contexts.

The cultural context of Poland, provides a timely and relevant setting in which to study parasocial relationships. Social media use in Poland has surged in recent years, and influencers have become prominent figures in the social landscape. For example, voices of people with disabilities who act as influencers on Polish social media are now making significant impact in public discourse. By sharing their experiences and advocating for accessibility in various tourist destinations, these influencers have built large followings and are influencing societal awareness and industry practice^28^, a role that arises through parasocial mechanisms of trust and identification with their audience. More broadly, contemporary Polish culture, much like Western culture, increasingly values authenticity and honesty in online content creators. Some researchers have noted a shift in consumer culture away from highly curated, “idealized” influencer images and toward more humanized and realistic portrayals of life^29^. Influencers who are perceived as genuine, relatable, and capable of building friendly relationships with their followers tend to be more successful in engaging Polish audiences. This trend underscores that parasocial relationships in Poland may hinge on similar factors (e.g., perceived authenticity and intimacy) as in other countries. The influence of influencers is not only social but also economic, their credibility and close parasocial connections with followers make them highly effective in marketing. Polish marketing research shows that campaigns involving popular influencers yield better results than traditional advertising, precisely because followers feel a personal bond and trust with the influencer. Indeed, Poland’s top influencers have turn parasocial capital into substantial financial gain, those with over a million followers can earn on the order of tens of thousands of zlotys per month^30^. These influencers have become symbols of success and aspiration for many young Poles, further fueling the cycle of followers investing emotionally in parasocial ties with them^30^. Despite the prominence of social media figures in Polish culture, there is little scientific information about the nature of parasocial relationships in Poland. Researchers and practitioners lack of a dedicated tools to measure parasocial phenomena among Polish users, making it difficult to investigate what motivates people to form these one-sided relationships and what effects such relationships might have in this cultural context. This gap highlights the importance of cross-cultural research and the adaptation of existing measures of parasocial engagement for use in Poland.

## The present study

To address this need, the present study aimed to adapt and validate the Multidimensional Measure of Parasocial Relationships (MMPR) in the Polish context. The MMPR has the potential of being a valuable tool for advancing research into parasocial relationships and their complex interplay with social media influencers^6^. As described earlier, the MMPR underwent rigorous psychometric testing in its original development. The scale demonstrated good internal consistency and construct validity. For instance, individuals with higher MMPR scores (i.e. more intense parasocial engagement) tended to report lower self-esteem, an effect that was statistically mediated by their higher propensity for social comparison with the media figure. This finding aligns with prior research on social media use and celebrity worship that links intense parasocial involvement to self-concept and well-being challenges. Altogether, the original MMPR proved to be a reliable and valid tool for capturing parasocial relational phenomena in its initial Swedish sample. However, as of yet its cross-cultural validity remains uneexplored. Indeed, as with much of the research on parasocial relationships, most studies have been conducted in Western contexts, such as Sweden and the United States. Cultural differences in media use, social norms, and relationship patterns could influence how parasocial relationships are experienced and reported. It was therefore essential to examine whether the MMPR’s factor structure would replicate in a different cultural setting.

In this study, we translated the MMPR into Polish and administered it to a sample of Polish adults to evaluate its psychometric properties. We expected to confirm the MMPR’s four-factor structure in Poland, reflecting the Affective, Behavioral, Cognitive, and Decisional dimensions observed in the original version. We also examined convergent validity by correlating MMPR scores with theoretically relevant variables, specifically the five early maladaptive schemas linked to interpersonal difficulties and measures of emotional well-being (i.e., positive and negative emotions). Based on the literature, our hypothesis was that stronger parasocial relationships would be associated with higher levels of maladaptive relational schemas (e.g., greater fears of abandonment, feelings of defectiveness) and with distinctive emotional profiles (for example, potentially higher negative emotion or loneliness if parasocial engagement compensates for unmet social needs, or conversely, possibly lower negative emotion if parasocial connections provide some emotional gratification)^31^. By adapting the MMPR to the Polish context and testing these predictions, this work not only provides a validated instrument for future studies in Poland but also contributes to the cross-cultural understanding of parasocial relationships. Ultimately, our introduction of a Polish version of the MMPR aims to facilitate further research into why and how Polish individuals form parasocial bonds, and what implications these bonds have for their well-being and social lives. See Table 1 for the MMPR in English and Polish.

## Methods

### Participants and procedure

The study included 371 participants from Poland, aged 18 to 48 years (*M* = 23.22, *SD* = 3.62). The sample comprised 255 women (68.7%) and 116 men (31.3%). Education levels varied: 135 participants held higher education (36.4%; 89 women and 46 men), 158 had secondary education (42.6%; 101 women and 57 men), 2 had vocational education (0.5%; 1 woman and 1 man), and 1 had primary education (0.3%; 1 man). Data were collected during 2023–2024 via online surveys administered on social media platforms (Facebook and Instagram). A snowball sampling strategy was used, with a survey link (created in LimeSurvey) distributed by the researchers through their personal networks, and recipients were asked to share the link further. Inclusion criteria required participants to be at least 18 years old and to follow at least one social media figure (e.g., influencer, YouTuber, etc.) with whom they have no real-life relationship. In addition, a subgroup of 34 young adults (ages 18–32 years, *M* = 20.97*, SD* = 2.263; 79.4% of women and 20.6% of men) completed the MMPR a second time after a 4-week interval for the assessment of test–retest reliability.

## Measures

### Multidimensional Measure of Parasocial Relationships (MMPR)

The MMPR assesses how and to what extent individuals engage in parasocial relationships. It is based on a multidimensional model of attitudes and evaluates four domains of parasocial engagement: emotions (Affective dimension), thoughts (Cognitive dimension), behaviors (Behavioral dimension), and decisions (Decisional dimension). The tool consists of 18 statements, each rated on a 4-point Likert scale (1 = *Strongly Disagree*, 4 = *Strongly Agree*). The four subscales are:

A. *Affective Dimension* (4 items): Captures emotional responses to a media personality and their content (e.g., feeling a sense of connectedness or emotional engagement when the media figure shares personal information). Example item: *I feel emotionally engaged when the media personality shares more personal information about themselves.*
B. *Behavioral Dimension* (4 items): Captures behaviors indicative of parasocial interaction, such as reading, viewing,“liking”, sharing, or commenting on the media personality’s posts. Example item: *I always like the posts of the media personality on social media.*
C. *Cognitive Dimension* (4 items): Captures thoughts and beliefs about the media personality, including attributed traits, values, and the perceptions of the media figure’s authenticity. Example item: *I believe the media personality represents values that are important to me.*
D. *Decisional Dimension* (6 items): Captures the extent to which the media personality is perceived to influence the individual’s everyday decisions. Example item: *I willingly follow various tips and advice shared by the media personality because I feel I can trust their expertise on the topic.*

A total MMPR score is calculated by summing all item responses, after reverse-coding where applicable, and dividing by 18, yielding an avarege score from 1 to 4. Subscale scores are computed similarly (i.e., sum of subscale items divided by number of items in that subscale). Higher scores indicate greater parasocial engagement. By convention, mean scores above 3.00 signify high engagement, between 2.00–2.99 indicate moderate engagement, and scores between 1.00–1.99 reflect low engagement.

### Young Schema Questionnaire – short form (YSQ)

Early maladaptive schemas were measured with the Polish adaptation of the Young Schema Questionnaire (short form)^32^. The full instrument contains 90 items assessing 18 schemas grouped into 4 domains: (1: Disconnection and Rejection, 2: Impaired Autonomy and Performance, 3: Excessive Responsibility and Standards, 4: Impaired Limits). Respondents rate items on a 6-point scale (1 = *Completely untrue*, 6 = *Describes me perfectly*), with higher scores indicating greater schema endorsement. In the present study, we administered a subset of 25 items covering five specific schemas (Emotional Deprivation, Abandonment, Defectiveness/Shame, Social Isolation, and Mistrust) identified in prior research as strongly associated with interpersonal problems. These schemas were chosen for their theoretical relevance to parasocial engagement (e.g., individuals high on abandonment or shame schemas may seek “safer” one-sided relationships). Other schemas were not included, as previous findings indicated they were less relevant or not significantly related to interpersonal problems in the context of virtual relationships. Example YSQ items are: “I worry that people I’m close to will leave or abandon me” (Abandonment schema) and “I feel that I’m inherently defective or inferior to others” (Defectiveness/Shame schema). Schema scores were computed as the mean of the relevant items.

### Scale of Positive and Negative Experience (SPANE)

Positive and negative emotional experiences were measured with the Polish adaptation of the SPANE^33^, also known as SPANE/SPANE-B and sometimes referred to as SPIND in Polish literature. The scale consists of 12 adjectives describing emotions (6 positive, 6 negative). Participants rate how often they experienced each emotion over the past four weeks on a 5-point scale (1 = *Very rarely or never*, 5 = *Very often or always*). The SPANE yields a Positive Experience score (sum of the 6 positive items), a Negative Experience score (sum of the 6 negative items), and an overall Affective Balance score (Positive Experience score minus Negative Experience score). Higher SPANE scores indicate more frequent positive or negative emotions, and a higher balance score indicates a more positive overall emotional experience. In this study we report the Positive, Negative, and Balance scores as convergent measures of well-being. Example items from SPANE include: “Joyful”, “Happy” (positive emotions) and “Sad”, “Afraid” (negative emotions).

## Translation and linguistic validation procedure

Written permission to adapt the scale was first obtained from the original authors. Prior to data collection, the MMPR was translated and culturally adapted into Polish following standard forward–backward translation procedures. The MMPR items were independently translated from English to Polish by three translators fluent in both languages. Five expert judges (advanced graduate students in clinical psychology, fluent in English) reviewed these translations to ensure conceptual equivalence; they selected or refined the phrasing that best captured the meaning of each original item. Next, a back-translation into English was conducted by an independent bilingual individual. The back-translation revealed no significant discrepancies, indicating that the Polish version accurately reflected the content of the original scale. The resulting Polish version was then piloted in a separate study to verify that the item content was clearly understood by participants. Minor wording adjustments were made based on this feedback to improve clarity. The finalized Polish version (see Table 1) was subsequently administered in the main study and subjected to psychometric analysis as described below.

## Results

### Descriptive statistics

Table 2 presents descriptive statistics for each dimension of the MMPR and the total score. The distribution of scores for the four dimensions significantly deviated from normality according to the Kolmogorov–Smirnov normality test (all *p* < 0.001), except for the overall MMPR score which was approximately normal. However, the skewness and kurtosis values for all variables were within acceptable range (−2 to + 2), indicating no severe departures from normality. Therefore, parametric tests were deemed suitable for further analyses. We also inspected item-level distributions, which showed that one item (item 6) showed high positive skewness (2.07), whereas skewness for the remaining items ranged from −0.66 (item 11) to 0.57 (item 8).

**Table 2:** Descriptive statistics for MMPR subscale and total score (*N* = 371)*.*

Dimensions	*Min*	*Max*	*M*	*Median*	*SD*	*Skewness*	*Kurtosis*	*K-S Z*	*p*	*α*	*ω*
Affective	1.00	4.00	2.47	2.50	0.62	−0.20	−0.35	0.11	< 0.001	0.65	0.67
Behavior	1.00	4.00	1.91	1.75	0.61	0.58	0.01	0.14	< 0.001	0.59	0.59
Cognitive	1.00	4.00	2.67	2.75	0.60	−0.14	0.12	0.10	< 0.001	0.73	0.73
Decisional	1.00	3.83	2.36	2.33	0.60	−0.24	−0.44	0.09	< 0.001	0.75	0.75
total score	1.11	3.72	2.35	2.39	0.45	0.07	0.11	0.04	0.16	0.83	0.83

*Note:* α = Cronbach’s alpha; ω = McDonald’s omega; K-S Z = Kolmogorov–Smirnov coefficient.

### Internal consistency

As seen in Table 2, the mean overall MMPR score was 2.35 (SD = 0.45) on the 1–4 scale, indicating moderate parasocial engagement on average among participants. Among the dimensions, the Cognitive dimension had the highest mean (2.67) and the Behavioral dimension the lowest (1.91). The internal consistency (Cronbach’s α) for the subscales ranged from α = 0.59 (Behavioral) to α = 0.75 (Decisional), with the Behavioral dimension showing the lowest reliability. McDonald’s omega values mirrored the alpha values for all dimensions. These reliability coefficients for the subscales were somewhat lower than those reported in the original MMPR development study. The total scale score showed good reliability (α = 0.83, ω = 0.83). We examined whether any single item’s removal would substantially increase the total scale’s reliability; no such item was identified, suggesting that all items contribute adequately to the overall scale.

### Confirmatory factor analysis (CFA)

**Fig. 1:**
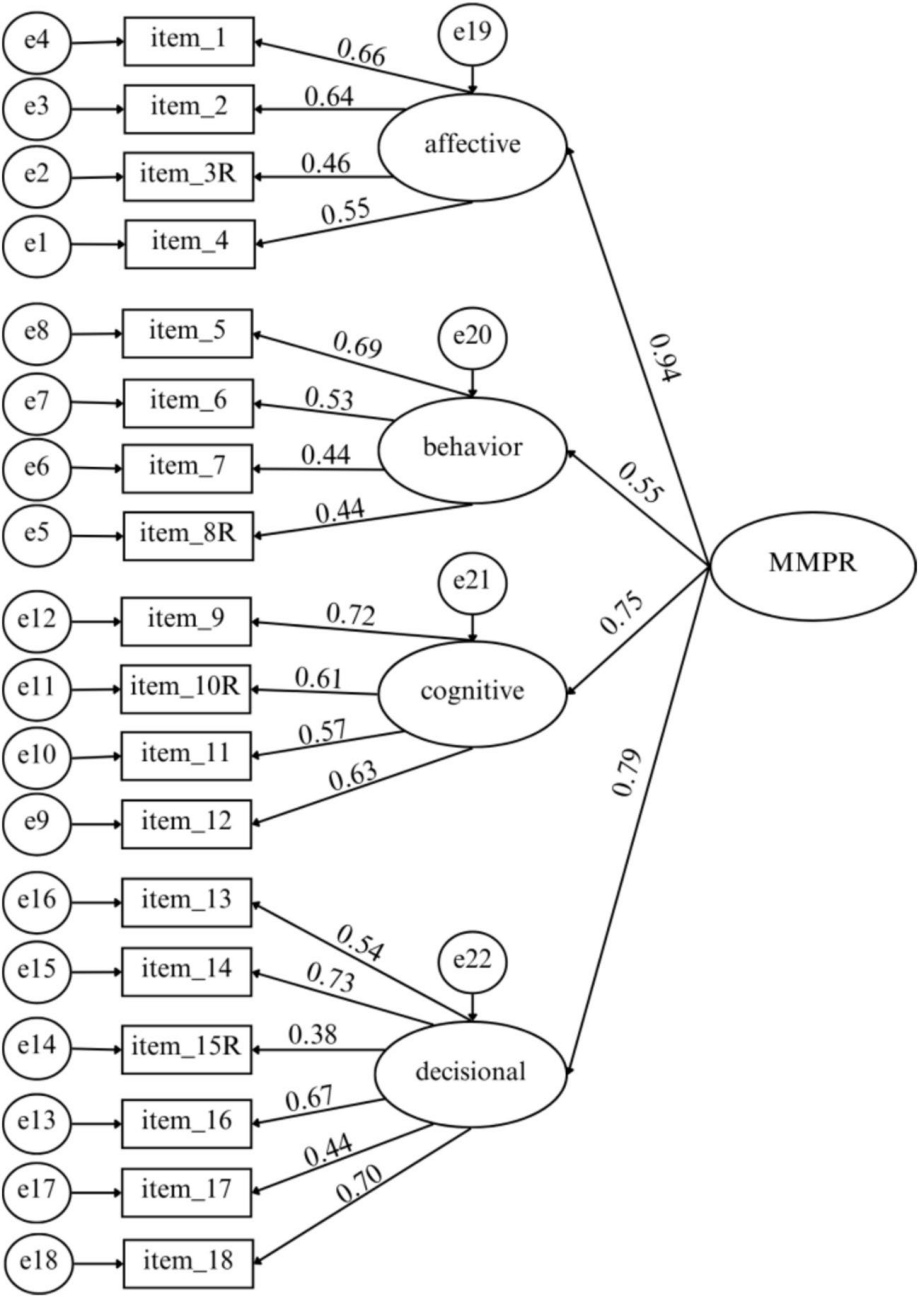
Path model of confirmatory analysis.

We conducted CFA to evaluate the MMPR-s factor structure in the Polish sample, using IBM SPSS Amos 29 with maximum likelihood estimation. A four-factor model (corresponding to the Affective, Behavioral, Cognitive, and Decisional dimensions) was specified and tested. All items’ loadings on their intended factors were statistically significant (see Fig. 1). Overall, the model fit the data adequately. The chi-square goodness-of-fit test was significant (χ^2^(131) = 358.81, p < 0.001), which is not unusual given the sample size and number of parameters. The relative chi-square (CMIN/df) was 2.74, falling within the range typically considered acceptable (between 2 and 3). Other fit indices also suggested an acceptable if not ideal fit: RMSEA = 0.069 (90% *CI* = [0.060- 0.077]), SRMR = 0.06, CFI = 0.855, and GFI = 0.899. The CFI and GFI values were slightly below the conventional cutoff of 0.90, which suggests the model is acceptable but marginal, warranting some caution in interpretation. In sum, the CFA provides support for the theorized four-dimensional structure of the MMPR in the Polish sample, albeit with room for improvement in model fit.

Inspection of the factor loadings revealed that all four latent dimensions were well-defined by their indicators, although the Behavioral dimension was the weakest of the four. Most standardized loadings ranged from 0.38 to 0.73. Notably, the Behavioral subscale items (items 5–8) had generally lower loadings (some below 0.60), consistent with the lower internal consistency observed for this subscale. In addition, item 15 (a reverse-worded item) in the Decisional dimension exhibited a particular low loading (λ ≈ 0.38). This low loading on a reverse-scored item may indicate that some respondents misunderstood the item or responded carelessly—a potential risk when collecting data on-line. We retained item 15 in the model to preserve content coverage, but this result suggests that the item may require rephrasing or further evaluation in future studies. Overall, despite these few low item loadings, the pattern of results supports the MMPR’s factorial validity. The four first-order factors (Affective, Behavioral, Cognitive, Decisional) all loaded significantly onto the overall parasocial engagement construct in the expected direction. We note that in this correlated-factor model, the inter-factor correlations ranged from 0.30 to 0.56 and conclude that the Polish version of the MMPR demonstrates an acceptable structural fit and captures the intended four-factor construct, although some measurement refinements (e.g., revising low-loading or reverse items, collecting additional data for invariance testing) could further strengthen the model.

### Test–retest reliability

As an estimate of temporal stability, the MMPR was re-administered after four weeks to a subsample of 34 young participants (79.4% women, 20.6% men). All Pearson correlation coefficients between Time 1 and Time 2 scores were positive, statistically significant, and ranged from moderate to high in magnitude. Specifically, stability coefficients were *r* = 0.473 for the Affective dimension (*p* = 0.005), *r* = 0.698 for the Behavioral dimension (*p* < 0.001), *r* = 0.723 for the Cognitive dimension (*p* < 0.001), and *r* = 0.809 for the Decisional dimension (*p* < 0.001). The test–retest correlation for the overall MMPR score was *r* = 0.781 (*p* < 0.001). These results indicated that the MMPR yields reliably stable scores over a one-month interval. However, caution is warranted in interpreting these reliability estimates because the retest sample was quite small and predominantly female. The lower stability of the Affective subscale might reflect the inherently fluctuating nature of emotions over time. In general, the moderate-to-high test–retest correlations suggest satisfactory temporal reliability of the scale in this initial evaluation.

### Convergent validity

Convergent validity of the MMPR was examined by correlating each parasocial relationship dimension and the total MMPR score with external constructs that theory suggest should be related to parasocial engagement. These constructs included the five selected maladaptive schemas and the emotional experiences measures (positive emotions, negative emotions, and affective balance from SPANE). Table 3 summarizes the Pearson correlation coefficients for these relationships.

**Table 3:** Pearson correlation coefficients between MMPR scores and external variables (convergent validity).

Scales	Variables	Parasocial relationship engagement (MMPR)				Total score MMPR
		Affective	Behavior	Cognitive	Decisional	
Early maladaptive schemas (YSQ)	Emotional Deprivation	−0.077	0.034	−0.078	−0.060	−0.062
	Abandonment	0.103	0.035	−0.001	0.151*	0.108
	Mistrust	0.022	0.111	−0.117	0.055	0.031
	Social Isolation	0.031	−0.062	−0.038	−0.045	−0.040
	Defectiveness/Shame	−0.017	0.127*	−0.076	0.066	0.040
Emotional experience (SPANE)	Positive Emotions	0.033	0.090	0.000	0.101	0.081
	Negative Emotions	−0.057	−0.206**	−0.072	−0.032	−0.114
	Affective Balance	0.052	0.169*	0.040	0.077	0.112

Note: Significant correlations in bold type. * Significance at the 0.05 level (two-tailed); ** Significance at the 0.01 level (two-tailed).

Several significant associations emerged. In particular, Behavioral parasocial engagement showed a positive correlation with the Defectiveness/Shame schema (*r* = 0.127, *p* < 0.05) and a negative correlation with negative emotional experiences (*r* = −0.206, *p* < 0.01). This pattern suggest that individuals who report more intense feelings of personal defect or unworthiness tend to engage more in parasocial behaviors, perhaps seeking affirmation or safe interaction, and that those who engage in more parasocial behaviors report fewer negative emotions. In practical terms, having negative self-beliefs might drive people to invest more in one-sided online relationships, and doing so could be associated with alleviating some negative emotional states, at least in the short term. Moreover, Decisional parasocial engagement was positively correlated with Abandonment schema (*r* = 0.151, *p* < 0.05). This implies that people who chronically fear abandonment or rejection in real-life relationships may be more inclined to allow media figures to influence their decisions, possibly because they find parasocial relationships “safer” or more controllable. The Affective and Cognitive dimensions of the MMPR showed no significant correlations with the measured schemas or emotion variables, and the total MMPR score likewise did not correlate significantly with these external measures (apart from nonsignificant trends in expected directions). The affective balance (overall emotional well-being) had a small positive correlation with Behavioral engagement (*r* = 0.169, *p* < 0.05), aligning with the observation that more parasocial activity was linked to fewer negative emotions.

In summary, the direction of the significant relationships was consistent with expectations and provides preliminary evidence of convergent validity for the MMPR. Parasocial engagement, especially behavioral and decisional aspects, was associated with certain early maladaptive schemas and emotion outcomes that make theoretical sense (e.g. higher parasocial involvement when one feels insecure in real relationships, and potential emotional benefits from parasocial activities). At the same time, many of the correlations were small or non-significant. The lack of stronger and broader correlations might indicate that parasocial relationship engagement in the Polish young adult context is a relatively distinct construct that is not heavily explained by general schemas or well-being measures. It is possible that other factors, such as specific personality traits, social media usage patterns, or motivations, mediate these relationships. This warrants further investigation, potentially using qualitative approaches or additional convergent measures, to deepen our understanding of what parasocial relationships mean for Polish young people and how they relate to psychological functioning.

## Discussion

The primary aim of this study was to adapt and validate the Multidimensional Parasocial Relationships Scale (MMPR) for use in Poland. Overall, the results indicate that the Polish version of the MMPR retains the intended four-factor structure (Affective, Behavioral, Cognitive, Decisional) and exhibits acceptable psychometric properties, with some qualifications. The confirmatory factor analysis (CFA) supported the four-dimensional model proposed in the original Swedish development study^6^. Key fit indices (CMIN/df, RMSEA, SRMR) suggested reasonable model fit for our data, although a couple of indices (CFI and GFI) fell just below conventional thresholds, suggesting the model is not a perfect representations of the Polish data. This is not uncommon in initial cross-cultural validations and signals an opportunity for further refinement of the model or scale. In particular, the Behavioral dimension emerged as a weaker point, showing the lowest internal consistency of the four subscales and its items tended to have lower factor loadings. Despite this, we chose to retain the Behavioral dimension subscale in the adaptation. It captures unique aspects of parasocial engagement (e.g., observable behaviors like viewing, liking, and sharing content) that are theoretically important to the construct’s multidimensional framework. Removing it could undermine the content validity of the scale. Future work should focus on improving this subscale, perhaps by rephrasing items (especially the reverse-scored ones) or adding new behavioral indicators, to achieve higher reliability without losing its conceptual coverage.

The reliability analyses in this study suggest that the MMPR is reasonably consistent and stable over time. The overall MMPR scale showed good internal consistency (α = 0.83), and the three other subscales (Affective, Cognitive, Decisional) had acceptable alpha values in the 0.65-0.75 range. The lower reliability of the Behavioral subscale, while concerning, may reflect the heterogeneity of parasocial behaviors or cultural differences in how these behaviors manifest. It is notable that the original MMPR study also found the Behavioral dimension to have the lowest alpha (around 0.66), which suggests this might be an intrinsic challenge with measuring the behavioral aspect of parasocial relationships. For instance, one person might passively consume a celebrity’s content, scoring high on viewing but low on interactive behaviors like commenting, whereas another might actively engage, leading to diverse response patterns that lower inter-item correlations. Moreover, the inclusion of a reverse-worded behavior item, which we found some evidence participants might misinterpret, can attenuate internal consistency if not all respondents understand it as intended. These factors should be explored in future studies.

Despite the lower alpha, the Behavioral subscale’s test–retest reliability was moderate (*r* ≈ 0.70 over 4 weeks), indicating that individuals’ behavioral engagement scores were fairly stable one month apart. Notably. The test–retest reliability for the other MMPR dimensions were strong, except for the Affective dimension which was moderate. This finding aligns with psychological literature suggesting that emotional states are more variable and context-dependent that traits or behaviors^34,35^. Overall, the evidence of stability, especially for the Behavioral, Cognitive, and Decisional dimensions and the total score, is encouraging, as it suggests the MMPR measures a construct that is not entirely momentary. That said, we acknowledge that the test–retest results should be interpreted with caution given the small retest sample and its skew toward young female participants. It would be beneficial for future research to replicate the test–retest assessment with a larger and more diverse sample to confirm these reliability estimates.

The pattern of convergent validity results provides some insight into the psychological correlates of parasocial engagement in our sample. We observed that higher parasocial behavioral engagement was associated with a greater tendency to feel defective or unworthy (Defectiveness/Shame schema) and with experiencing fewer negative emotions. Meanwhile, greater decisional influence by media figures correlated with higher fear of abandonment. These findings fit a theoretical narrative: individuals who struggle with self-esteem or fear rejection might find parasocial relationships especially appealing because these one-sided relationships offer emotional safety and a sense of connection without the risks inherent in mutual relationships. For such individuals, engaging in parasocial behaviors (e.g., liking, commenting, etc.) might provide a form of compensatory affection or validation, potentially improve mood or at least distraction from negative feelings. In line with this, the negative correlation between parasocial activity and negative emotional experience hints that parasocial engagement could play a role in emotion regulation, serving as an outlet or coping mechanism to alleviate loneliness or sadness. This interpretation resonates with prior research suggesting that parasocial relationships can buffer feelings of social rejection and provide emotional relief for those lacking real-life social support^36–39^. However, contrary to appearances, such relationships do not promote the enhancement of self-worth but may only deepen beliefs about one’s own imperfection and inability to form lasting, authentic connections. Furthermore, identifying with idealized media figures within parasocial relationships can lead to a stronger sense of personal inadequacy^40^. Comparing oneself to such images can reinforce schemas of defect and shame. In this context, unrealistic expectations regarding these types of relationships can intensify the fear of rejection in real interactions, as the individual may develop unrealistic standards for what a true interpersonal relationship should look like^11^.

On the other hand, it is equally important to note the limits of these associations. Many expected correlations (e.g., between parasocial engagement and the other maladaptive schemas, or between engagement and positive emotions) were weak or non-significant. This could imply that parasocial relationship involvement is not strongly driven by broad personality or well-being factors, but rather by more specific motivations or situational factors (e.g., media usage habits, identification with certain influencers, etc.). Another interpretation is that parasocial relationships among young Polish adults might carry different meanings or consequences than in other contexts. For example, the generally low correlations might indicate that, in Poland, parasocial engagement is a more normative social behavior, part of everyday media consumption, rather than a compensatory mechanism for psychological issues—unlike what might be observed in other cultures or older cohorts. This points to the need for further research, possibly qualitative interviews or cross-cultural comparisons, to understand the subjective experience and nuances of parasocial relationships in this demographic. Given the increasing importance of parasocial relationships in society^11,24,41–43^, the MMPR is particularly relevant for this endeavor.

### Limitation and strengths

Like all studies, this work has limitations that must be acknowledged. A notable limitation is the sampling method and sample composition. Our use of convenience sampling via media may introduce sampling vias. The sample was predominantly young adults, especially women in their early twenties who are active social media users. As a result, the findings may not generalize to other age groups or to individuals who are not regular social media consumers. Older adults or those with limited internet access are underrepresented, which could limit the applicability of the MMPR in those populations. Additionally, cultural context plays a significant role in parasocial phenomena; our results are specific to Poland’s social media environment and cultural norms. Caution should be taken in assuming the MMPR would perform similarly in markedly different cultural settings without further validation. For instance, more collectivist cultures might emphasize community values in parasocial bonds, whereas our primarily individualistic Polish sample may focus more on personal identification with media figures. Future studies should aim for more diverse and representative samples (e.g., in terms of age, gender, and background) and could test measurement invariance across cultures to ensure the tool’s broader applicability.

Another limitation is related to the convergent validity outcomes. The relatively small or null correlations with schemas and well-being measures raise questions about whether the MMPR captures a distinct construct or if our chosen validation measures were suboptimal. It’s possible that parasocial engagement’s links to psychological factors more indirect or moderated by other variables (such as underlying personality traits, loneliness levels, or specific media contexts). The cross-sectional design of our study precludes any causal inferences—whether parasocial engagement influences well-being or vice versa cannot be determined. Longitudinal designs or experimental studies would be needed to untangle the direction of effects (e.g., whether engaging in parasocial relationships improves well-being by providing pseudo-social support, or whether people who are happier simply engage more positively with media figures). We also acknowledge that self-report measures like the MMPR are susceptible to biases such as social desirability or inaccurate self-perception. Participants might under-report behaviors that they think are unfashionable (e.g., “obsessive” fandom) or over-report positive feelings to align with social norms. Incorporating behavioral data (e.g., actual social media usage metrics) or informant reports could provide a more objective complement to self-reported parasocial engagement in future research.

Measurement-wise, although the overall factor structure was supported, some fit indices and item statistics were not ideal. In particular, the CFA showed a CFI slightly below the preferred threshold of 0.90, and one reversed item (item 15) had a very low factor loading. These issues suggest there is room for improvement in the scale’s psychometric robustness. Refining or clarifying problematic items, especially reverse-worded items that may confuse respondents taking the survey online, could enhance the internal consistency and model fit. The Behavioral subscale, while conceptually important, had lower reliability, which might stem from the wording of items or cultural differences in expressing parasocial behaviors. Future adaptation efforts might consider adding a couple of new behavior items or adjusting existing ones to better capture the range of parasocial activities in the local context. For example, behaviors like following an influencer’s live streams or engaging in fan discussions might be relevant in Poland and could be included to broaden the subscale’s scope.

Despite these limitations, our study also has notable strengths. To our knowledge, this is the first attempt to culturally adapt and validate the MMPR outside of its original context, contributing to the understanding of parasocial relationships in a non-English-speaking population. We employed a rigorous adaptation proces, including translation, expert review, and back-translation, to ensure linguistic and conceptual equivalence of the instrument. The sample size for the main survey (*N* = 371) was adequate for factor analysis and reliability testing, providing a solid initial test of the scale’s properties. We also incorporated multiple forms of reliability and validity evidence (i.e., internal consistency, test–retest stability, CFA for structural validity, and correlations with theoretically relevant constructs) to build a comprehensive validity argument for the Polish version of the MMPR. Furthermore, by examining both positive and negative outcomes (i.e., well-being indicators and maladaptive schemas), we shed light on the dual nature of parasocial engagement, its potential benefits and risks, in line with current debates in the literature. These strengths bolster confidence in our findings and demonstrate the value of the MMPR as a multidimensional tool for parasocial relationship research.

## Conclusion

The Polish adaptation of the MMPR appears to be a psychometrically sound instrument for assessing parasocial relationships across emotional, behavioral, cognitive, and decisional domains. By integrating the concept of parasocial interactions (i.e., momentary, one-sided engagements) with parasocial relationships (i.e., enduring bonds with media figures) into a single measure, the MMPR provides a comprehensive framework to examine how people engage with public figures in the social media age. This holistic approach is advantageous because repeated parasocial interactions (e.g., liking posts, watching daily stories, etc.) often cumulate into a perceived relationship over time. The MMPR’s multidimensional structure allows researchers to capture not only how strongly individuals feel connected to media personalities, but also how these figures influence users’ behaviors and decisions in daily life. Our findings highlight that parasocial engagement is a complex phenomenon with both positive facets (e.g., potentially mitigating negative emotions or providing a sense of belonging) and negative facets (e.g., possibly reflecting unmet needs or maladaptive coping for those high in abandonment fears). Cross-cultural validation efforts like this study are crucial. They ensure that constructs originally studied in one cultural milieu (in this case, Sweden) are applicable and relevant in others (here, Poland). The present research opens the door for further investigations into parasocial relationships in Poland, where social media influencers are increasingly prominent in the public sphere, yet empirical studies on audience motives and effects are still scarce. Our successful adaptation of the MMPR lays the groundwork for such studies. Encouragingly, parallel projects are underway to adapt the MMPR in several other countries, including Brazil, the Philippines, Nigeria, Colombia, El Salvador, India, Lithuania, Malaysia, and Indonesia, which will facilitate cross-cultural comparisons. In conclusion, the adapted MMPR is a valuable instrument for advancing parasocial relationship research in Poland and beyond, enabling scholars to better understand how one-sided connections with media figures impact individuals’ social and emotional lives across different cultural contexts.

*"Każdy z nas jest tłumem"[Each of us is a crowd] – Wisława Szymborska*

## Data Availability

The data supporting the findings of this study are available from the research group, but restrictions apply to the availability of these data, and the data are not publicly available. In case data is requested, please contact D.G.
